# Construction Notes

**Published:** 1895-09-14

**Authors:** 


### CONSTRUCTION NOTES.

### Convalescent Hospital at Woodthorpe.

This home was formally opened last month by the Bishop of Derby; it is in connection with the Nottingham General Hospital. This is the latest gift of Colonel Seeley, M.P., to the parent institution, who purchased The Cedars at a cost of £6,000 and transferred it to the trustees of the General Hospital. One half of this building is set apart for women and the other half for men patients, eight beds being provided for each, with two additional ones on the men's side for children. Each portion of the building has its own staircase, an additional one communicating with the servants' apartments. There is a good-sized room for both sexes. The matron and nurse in charge have separate sitting-rooms on the ground floor. The architects for the structural alterations are Messrs. Brewill and Baily, of Nottingham.

### Tiverton and District Isolation Hospital.

The Report of the Joint Committee of the Tiverton Town Council and Tiverton Rural District Council on the proposed erection of an isolation hospital for the town and country has been prepared. The site is about two miles from the centre of Tiverton, on the Halberton Road, and consists of three acres. Unlike the contention which usually is presented in the choice of site for an infectious diseases hospital, this, from the first, was considered an admirable one, being 350 feet above sea level, and well placed as regards immediate surroundings, providing every facility for drainage, and being easily accessible. The accommodation here to be provided consists of eight beds in the south pavilion for scarlet fever patients, eight beds in the north pavilion for diphtheria and fevers other than scarlet fever, two beds in the emergency ward, and two beds on the second floor of the administrative block for private patients. There will be various detached buildings; the closets will be on the dry earth system, and the drainage will be taken to the north corner of the site, and there dealt with by means of subsoil irrigation. The method of planning here adopted by Mr. Siddalls, the architect, is such that the hospital may be enlarged when required in a simple and inexpensive manner by extending the ward pavilions without in any way interfering with the general harmony of the design. The estimate of the total cost is about £3,600.

### Children's Ward to Bideford and District Infirmary.

The last two years have seen a decided increase in the number of children patients at this infirmary. As, therefore, the number of beds had to be augmented, the governors invited the public to help them to build and furnish a children's ward. The response was immediate and gratifying, and Mr. Hookway was instructed to prepare plans. He converted the committee-room on the south side into a children's ward of six beds, designing another committee-room on the north side. The extension here enabled an addition to be made to the out-patients' waiting-room below, doubling its accommodation, a bed-room for the additional nurse engaged for the children's ward being reserved upstairs. The cost of all this work, carried out by Mr. E. Ellis, contractor, was £683; the total cost, including architect's fees, &c., being £677.

### Grimsby and District Hospital.—Improvements and Alterations.

Mr. Scaping, the hospital architect, has presented plans for the following alterations in this institution. A servants' hall is to be constructed on the left-hand side of the ground floor central entrance. On the right-hand side of the same a large store-room is to be constructed, all party walls between them being taken out. On the first floor level, in the corridor, it is proposed to build a new operating theatre, attached to which will be an anæsthetic-room. Close at hand, also, will be a new nurses' sitting-room, and large drying store for household requisites. These alterations are planned to be commenced in the spring.

